# Editorial: Tumor microenvironment (TME) and tumor immune microenvironment (TIME): New perspectives for prognosis and therapy

**DOI:** 10.3389/fcell.2022.971275

**Published:** 2022-08-22

**Authors:** Rodrigo Nalio Ramos, Mariane Tami Amano, Adriana Franco Paes Leme, Jay Willian Fox, Ana Karina de Oliveira

**Affiliations:** ^1^ Departament of Hematology and Cell Therapy, Laboratory of Medical Investigation in Pathogenesis and Directed Therapy in Onco-Immuno-Hematology (LIM-31), D’Or Institute for Research and Education (IDOR), Hospital Das Clínicas da Faculdade de Medicina da Universidade de São Paulo, São Paulo, Brazil; ^2^ Hospital Sirio Libanes, São Paulo, Brazil; ^3^ Brazilian Biosciences National Laboratory (LNBio), Brazilian Center for Research in Energy and Materials (CNPEM), Campinas, Brazil; ^4^ Department of Microbiology, Immunology and Cancer Biology, Office of Research Core Administration (ORCA), University of Virginia School of Medicine, Charlottesville, VA, United States; ^5^ Department of Pathology, University of Virginia School of Medicine, Spatial Biology Core (SBC) Facility, Charlottesville, VA, United States

**Keywords:** tumormicroenvironment, tumor immune microenvioronment, immune checkpoint blockade, immune therapeutics, tumor stroma cross-talk, prognosis

The success of tumor treatment is defined by a combination of factors that include the cells that make up the tumor microenvironment (TME). TME studies have contributed to illuminating the heterogeneity of tumors and has led to the discovery of potential targets for cancer immunotherapeutics. New strategies are continually being based on these studies are being approved for clinical use at a greater rate than compared in the past decade. In the 2000s, the first studies of the effects of PD-1, PDL-1, and CTLA-4 inhibitors started, and currently, there are dozens of promising immunotherapy targets in clinical trials studies or already available in the clinical practice, including genetically modified T cells expressing chimeric antigen receptors (CAR-T cells), anti-CD20 (Rituximab) for treatment of non-Hodgkin’s B cell lymphoma, anti-CD52 (Alemtuzumab) for multiple myeloma, anti-IL-6 (Siltuximab) and the anti-IL-6R (Tocilizumab) for multiple myeloma and solid tumors, anti-LAG3 (Relatlimab) immune activating checkpoints in melanoma.

Although great advances have been made in the immunotherapeutic field, only about 30% of patients respond to these treatments. In some cases, tumors acquire resistance to immune checkpoint blockade (ICB) by losing neoantigen expression or even presenting a low frequency of T cell infiltration, or other unknown reason. Investigators still have a long way to go to improve strategies against cancer that including the discovery of new targets and strategies to activate the immune system, the modulation of phenotype and function of cells present in the TME, the re-education of immune cell function, and the induction of immune cell migration to the tumor site.

**FIGURE 1:**
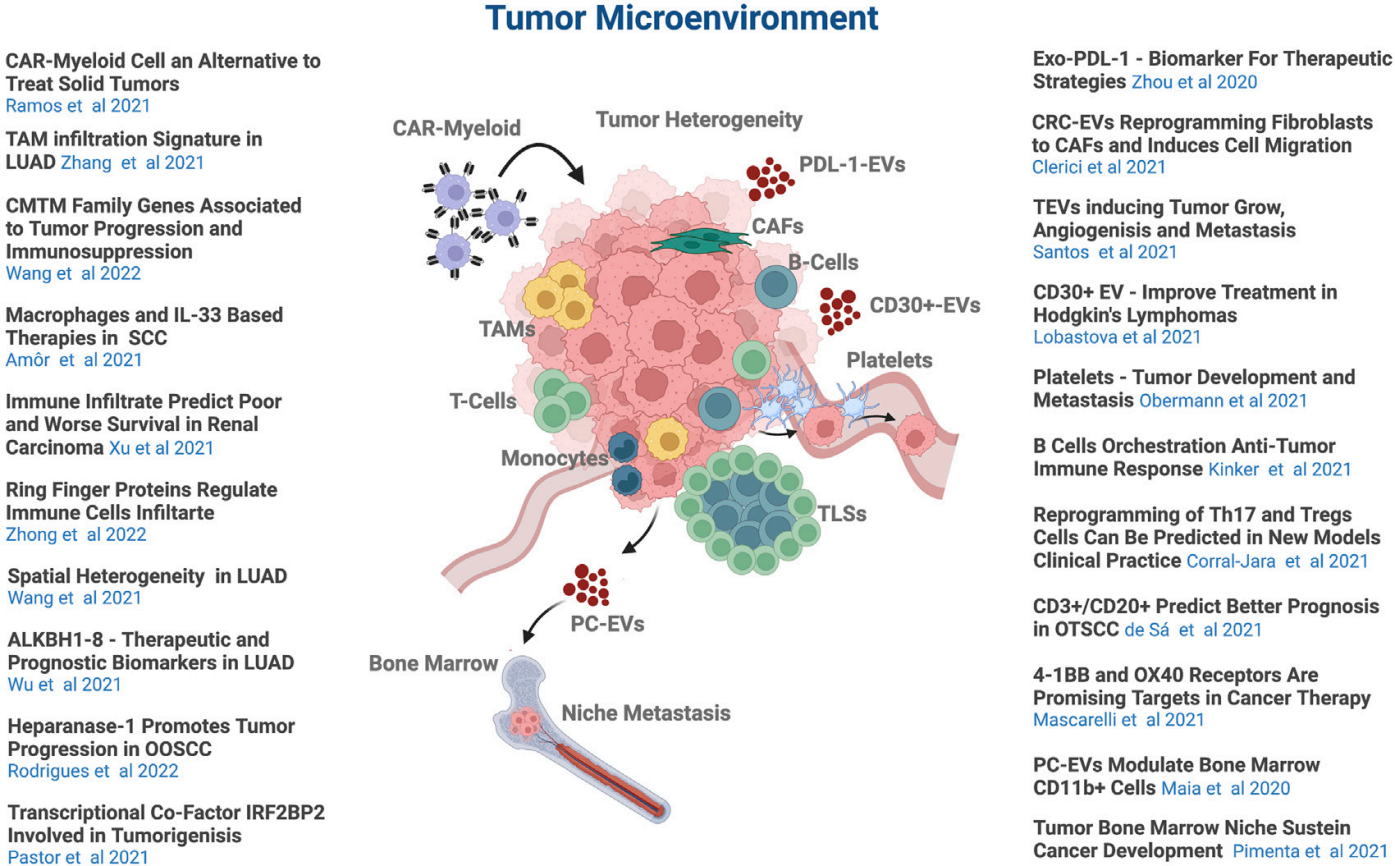
The tumor microenvironment. A schematic view of the tumor microenvironment components and all scientific contributions in this Topic Research. The picture was created with BioRender.com.

This Research Topic presents a remarkable collection of articles comprised of 9 Original Research, 2 Brief Research Reports, 8 Reviews, and 2 Mini Reviews covering novel, promising recent trends in the immune microenvironment landscape and immunotherapy field (Figure 1).

The TME is a complex ecosystem where continuous interactions sustain tumor development and growth. Recent molecular biology and immunology findings have shown that tumor-stroma communication promotes intense suppressive signals in the microenvironment, contributing to therapy failure. Tumor-stroma communication is not a unidirectional process, driven only by cancer cells. Other resident cells, like cancer-associated fibroblasts (CAFs), macrophages, lymphocytes, endothelial cells, and cancer stem cells, have intense crosstalk with each other, with secreted factors and components of the extracellular matrix (ECM). New technologies have been emerging to map these ecosystems. Machine learning (ML) is one of these promising technologies that has revolutionized this area of research. Big data combined with ML have changed many aspects of the experimental domain and may ultimately support the clinical decision process in cancer therapy.

In this context, Zhang et al. used bioinformatics approaches to build a tumor-associated macrophage (TAM) prognostic risk model. They generated a relevant gene model correlated with clinical features and predictive risk scores and thereby determined a central regulatory axis: LINC00324/miR-9-5p (miR-33b-5p)/GAB3 (IKZF1), which may play a pivotal role in regulating the risk and prognosis of TAM in patients with lung adenocarcinoma (LUAD). Using a training and validation cohort model with more than 900 patients from different databases, Wang et al. constructed a score based on the cluster model. This model was able to stratify lower-grade glioma (LGG) patients into two groups concerning the chemokine-like factor (CKLF)-like Marvel transmembrane domain-containing (CMTM) gene family: low-risk score groups associated with high tumor purity and reduced immune cell infiltration; and the high-risk score group which exhibited a poor prognosis with higher grade wild-type isocitrate dehydrogenase (WT-IDH), high expression of CMTM genes, and increased expression levels of PD-1, PD-L1, and PD-L2.

Considering the remarkable role of extracellular vesicles (EVs) in cell-to-cell communication in the TME and the emergent number of novel studies in literature, it is no surprise that five articles in this thematic field were published on this topic. EVs can originate from either endosomal (exosome) or plasma membrane budding (microvesicles). Some have been demonstrated to have a pro-tumoral activity in the TME, impacting cancer cell proliferation, immune escape, angiogenesis, invasion, pre-metastatic niche formation, and metastasis. Based on these characteristics they may become important therapeutic targets and prognostic biomarkers as was discussed by Santos et al. in their review. Clerici et al. demonstrated another aspect of colorectal cancer-derived EVs, which reprogram fibroblasts, induce migration, and contribute to the drug resistance phenotype.

Interestingly, Maia et al. show communication between bone marrow CD11b+ and pancreatic tumor cells mediated by EVs. This communication induces genetic alterations in the TME, such as macrophage activation and expression of inflammatory molecules. Zhou et al. show the predictive potential of tumor cell-derived exosomal PD-L1+ as a marker of immunotherapeutic efficacy, while Lobastova et al. show that CD30^+^ EVs can increase the treatment efficacy of Hodgkin’s lymphomas.

Kinker et al. in their comprehensive review of tertiary lymphoid structures (TLS), highlighted the TLS structure in the TME. The TLS provides an area of intense B cell antigen presentation that induces T cell activation and effector B cell generation, which induces the antibody-secreting plasma cells and memory B cells differentiation. The TLS is a well-organized non-encapsulated structure composed of immune and stromal cells, which are associated with improved response to immune checkpoint therapies. Evaluating patients with Oral Tongue Squamous Cell Carcinoma, Sales de Sá et al. show that high expression of CD3^+^ T cells and B cells in TLS regions are predictive of better overall survival and inflammatory response.

A number of articles discussed the participation of specific molecules in tumorigenesis and cancer metastasis. Obermann et al. reviewed the role of platelets in the formation of micrometastases and the formation of the metastatic niche. Indeed, metastatic niches seem to contribute exponentially to tumor progression. Different niches present in the bone marrow (BM) are altered during acute myeloid leukemia (AML) development. These niches that support the development of AML are also important targets for the development of future therapies since these targets are involved in critical functions of leukemia (Pimenta et al.). Pastor et al. described the involvement of Interferon regulatory factor 2-binding protein 2 (IRF2BP2) in tumor development. The role of heparinase-1 in oral squamous cell carcinoma tumor progression was investigated by Rodrigues et al.

In the domain of the emerging therapies, Ramos et al. provided a magnificent review of the new approaches of Chimeric antigen receptor (CAR) engineering in solid tumor treatment using CAR-myeloid cells. CAR therapy is an already established technology used for T cells (CAR-T) and natural killer cells (CAR-NK). In CAR T-cell therapies, whereby the patient’s T-cells are genetically reprogramed to express a chimeric antigen receptor thereby driving T cells to eliminate tumors. CAR-T is used in hematologic tumors but has yet to demonstrate overall efficacy in solid tumors. Thus, the use of genetically modified macrophages and dendritic cells expressing CAR (CAR-myeloid cells) may open new perspectives in treating solid tumors.

We are confident that our Tumor Microenvironment (TME) and Tumor Immune Microenvironment (TIME): New Perspectives for Prognosis and Therapy Research Topic has highlighted important aspects of tumor microenvironment heterogeneity, new therapeutic and prognostic targets, interactions in TEM, new technologies for the study of cancer, and more importantly, and also indicated that there is yet a long path forward to provide robust cancer prognoses and therapies in cancer.

